# Characterization of Microsporidia-Induced Developmental Arrest and a Transmembrane Leucine-Rich Repeat Protein in *Caenorhabditis elegans*

**DOI:** 10.1371/journal.pone.0124065

**Published:** 2015-04-13

**Authors:** Robert J. Luallen, Malina A. Bakowski, Emily R. Troemel

**Affiliations:** Division of Biological Sciences, Section of Cell and Developmental Biology, University of California San Diego, La Jolla, California, United States of America; Louisiana State University, UNITED STATES

## Abstract

Microsporidia comprise a highly diverged phylum of intracellular, eukaryotic pathogens, with some species able to cause life-threatening illnesses in immunocompromised patients. To better understand microsporidian infection in animals, we study infection of the genetic model organism *Caenorhabditis elegans* and a species of microsporidia, *Nematocida parisii*, which infects *Caenorhabditis* nematodes in the wild. We conducted a targeted RNAi screen for host *C*. *elegans* genes important for infection and growth of *N*. *parisii*, using nematode larval arrest as an assay for infection. Here, we present the results of this RNAi screen, and our analyses on one of the RNAi hits from the screen that was ultimately not corroborated by loss of function mutants. This hit was an RNAi clone against *F56A8*.*3*, a conserved gene that encodes a transmembrane protein containing leucine-rich repeats (LRRs), a domain found in numerous pathogen receptors from other systems. This RNAi clone caused *C*. *elegans* to be resistant to infection by *N*. *parisii*, leading to reduced larval arrest and lower pathogen load. Characterization of the endogenous F56A8.3 protein revealed that it is expressed in the intestine, localized to the membrane around lysosome-related organelles (LROs), and exists in two different protein isoforms in *C*. *elegans*. We used the CRISPR-Cas9 system to edit the *F56A8*.*3* locus and created both a frameshift mutant resulting in a truncated protein and a complete knockout mutant. Neither of these mutants was able to recapitulate the infection phenotypes of the RNAi clone, indicating that the RNAi-mediated phenotypes are due to an off-target effect of the RNAi clone. Nevertheless, this study describes microsporidia-induced developmental arrest in *C*. *elegans*, presents results from an RNAi screen for host genes important for microsporidian infection, and characterizes aspects of the conserved *F56A8*.*3* gene and its protein product.

## Introduction

Microsporidia represent a large phylum of obligate intracellular pathogens that are related to fungi, with significantly reduced genomes compared to true fungi and other eukaryotes [1–4]. There are 14 species of microsporidia that can infect humans, and this can lead to an invasive infection that is sometimes lethal when host immunity has declined, as in patients with AIDS or those on immunosuppressant therapy [5]. Microsporidia can also be isolated from asymptomatic immunocompetent people, with reports finding up to 56% of this population shedding microsporidian spores [6]. Most species found in humans infect the intestine, including *Enterocytozoon bieneusi*, *Encephalitozoon cuniculi*, and *Encephalitozoon intestinalis* [7]. Very little is known about microsporidian mechanisms of pathogenesis due to the difficulties of culturing these microbes.

We use the nematode *Caenorhabditis elegans* as a convenient, whole-animal system to study microsporidian infection. In its natural environment, *Caenorhabditis* nematodes are regularly infected by microsporidia, and we focus on a microsporidian species isolated from wild-caught *C*. *elegans* found in a compost pit near Paris [8–10]. This organism, named *Nematocida parisii*, infects *C*. *elegans* intestinal cells where it undergoes extensive replication that eventually leads to death of the host. Due to the many genetic tools available in *C*. *elegans*, we use *N*. *parisii* infection of *C*. *elegans* as a model for discovery-based genetic screens to find host genes important for microsporidian infection and progression.

Here, we present the results of a screen for host genes important for infection. We also present our analysis of the gene corresponding to an RNAi hit from the screen that was ultimately not corroborated by loss of function mutations in that gene. This screen involved searching for *C*. *elegans* RNAi clones that block infection, measured as a reduction in the severity of *N*. *parisii*-induced larval arrest. We chose to focus on one RNAi clone discovered in this screen based on its robust and specific inhibition of infection-induced larval arrest, and the identity of its target gene, *F56A8*.*3*, which encodes a predicted transmembrane domain protein with leucine-rich repeats (LRRs), which are found in many pathogen receptors. We found that feeding *C*. *elegans* the *F56A8*.*3* RNAi clone resulted in lower *N*. *parisii* pathogen load at various stages of infection, and that endogenous F56A8.3 protein localized to the membranes around lysosome-related organelles (LROs). However, after mutating *F56A8*.*3* using targeted genome editing with the CRISPR-Cas9 system, we found that mutations in *F56A8*.*3* did not recapitulate the infection phenotypes of the RNAi clone, indicating that these phenotypes are due to an off-target effect of the clone. The results described here provide new information about a microsporidian infection-induced phenotype in *C*. *elegans*, and the characterization of a LRR protein in *C*. *elegans* that shows conservation in other animals.

## Materials and Methods

### *C*. *elegans* and *N*. *parisii* culture conditions

All *C*. *elegans* strains were maintained on nematode growth media (NGM) and fed with *E*. *coli* strain OP50-1, as described [11]. *N*. *parisii* spores were prepared as previously described [12]. Briefly, *N*. *parisii* isolate ERTm1 was cultured by infecting large-scale cultures of *C*. *elegans*, followed by mechanical disruption of the nematodes and then filtering to isolate spores away from animal debris. The RNAi-sensitive sterile strain GR1373 *eri-1(mg366)* was used for the larval arrest screen and subsequent RNAi experiments [13]. The tissue-specific RNAi strains MGH167 *sid-1(qt9); alxIs9 [VHA-6p*::*SID-1*::*SL2*::*GFP]* and SPC272 *sid-1(qt9); Is[myo-3*::*sid-1]* were kind gifts from Drs. Gary Ruvkun, Justine Melo, Sean Curran, Antony Jose, and Alex Soukas [14, 15], WU1236 *cdf-2(tm788); amIs4[cdf-2*::*GFP*::*unc-119(+))]* was a kind gift from Dr. Kerry Kornfeld, and GH351 *glo-3(zu446) X; kxEx41[glo-3p*::*glo-3*::*GFP; rol-6]* was a kind gift from Greg Herman [16, 17]. Two *F56A8*.*3* promoter strains ERT173 *jyEx77[pF56A8*.*3a*::*mCherry*::*unc54 3’UTR]* and ERT174 *jyEx78[pF56A8*.*3a*::*mCherry*::*unc54 3’UTR]* were generated for this study (see cloning details below). *F56A8*.*3* mutant strains ERT327 *F56A8*.*3(jy4)* and ERT425 *F56A8*.*3(jy8[LoxP Prps-0*::*HygR-CeOpt*::*gpd-2*::*GFP*::*unc-54 3’UTR LoxP)* were generated by CRISPR-Cas9 and backcrossed three times to N2, and these strains were crossed to GR1373 *eri-1(mg366)* to make ERT360 *F56A8*.*3(jy4); eri-1(mg366)* and ERT430 *F56A8*.*3(jy8[LoxP Prps-0*::*HygR-CeOpt*::*gpd-2*::*GFP*::*unc-54 3’UTR LoxP); eri-1(mg366)*, respectively.

### Larval arrest experiments

For the larval arrest screen, cherry-picked RNAi libraries derived from the *C*. *elegans* Ahringer feeding RNAi library were used, which included approximately 345 RNAi clones for predicted transcription factors and 91 RNAi clones for LRR genes [18]. Conditions for the screen were modified from published procedures [19]. Specifically, RNAi clones were amplified and plated on RNAi plates (6-well format) in duplicate overnight at 25°C. Five synchronized L1 *eri-1* animals were hand-picked onto each RNAi clone and grown for 65–66 hours at 20°C until hundreds of F1 generation L1s and eggs were observed. Wells were infected with *N*. *parisii* spores at 5.5 x 10^6^ spores in 200 μl M9 per well and shifted to 25°C, which causes sterility in *eri-1* mutants and prevents further reproduction. At 2 days post-infection (dpi), the infected F1 generation animals in each well were visually scored together by overall size on a 1–4 scale. Completely unarrested animals that reached the young adult stage were scored as a 4, similar to uninfected *eri-1* grown on L4440 (control RNAi). Wells with partially arrested animals where the majority of animals reached the L4 larval stage were scored as a 3. Wells with more severely arrested animals, with the majority of animals in the L2 or L3 larval stage were scored as a 2. Fully arrested wells where the majority of animals were still at the L1 larval stage were scored as a 1. Because *eri-1* mutants are sterile at 25°C, there were no F2 generation animals to affect the assay. Larval arrest assays performed on hits involved scoring 100 animals per replicate (conducted in triplicate per experiment) that reached the L4 or adult stage at 2 or 3 dpi, respectively, using strains *eri-1*, MGH167, SPC272, ERT360, and ERT430.

For larval arrest experiments on *P*. *aeruginosa* (PA14), synchronized *eri-1* mutants were grown for one generation on RNAi from L1 to adulthood, and then bleached to obtain F1 eggs. SK plates were seeded with 20 μL of overnight cultures of PA14 and incubated at 37°C for 24 hours, followed by 25°C for 24 hours. A total of 100 RNAi-treated F1 eggs obtained from bleach were plated in duplicate on PA14 plates at 25°C, and the number of hatched animals reaching the L4 stage at 2 dpi was recorded.

### Pathogen load measurement

For pathogen load measurements by quantitative RT-PCR (qRT-PCR), *eri-1* mutants were treated with RNAi for two generations. Specifically, gravid *eri-1* adults were bleached to obtain starved L1s. These synchronized P0 L1s were then grown to gravid adults on RNAi bacteria and bleached again to obtain synchronized F1 progeny, which were plated on fresh RNAi bacteria. These F1 L1 progeny were infected with a two-fold serial dilution of *N*. *parisii* starting at 5.76 x 10^6^ spores per 10 cm plate at 25°C. At 30 hours post infection (hpi), animals were harvested and RNA was isolated by extraction with Tri-Reagent and bromochloropropane (BCP) (Molecular Research Center). cDNA was synthesized from 250–500 ng of RNA with the RETROscript kit (Ambion) and quantified with iQ SYBR Green Supermix (Bio-Rad) on a CFX Connect Real-time PCR Detection System (Bio-Rad). Pathogen load was measured as the relative abundance of an *N*. *parisii* ribosomal DNA (rDNA) transcript normalized to a *C*. *elegans* rDNA transcript with the following primer sets: Np_rDNAF1: aaaaggcaccaggttgattc, Np_rDNAR1: agctctctgacgcttccttc, Ce18S_F1: ttgcgtacggctcattagag, Ce18S_R1: agctccagtatttccgcagt. Primer efficiencies were measured, and fold difference was calculated with using the Pfaffl method [20].

For pathogen load measurement by fluorescence in situ hybridization (FISH) experiments were performed essentially as described [21]. Specifically, *eri-1* animals were treated on duplicate plates with RNAi as above, but F1 progeny were grown for 24 hrs at 20°C before infection with 2.9 x 10^6^ spores per 10 cm plate for 8 hours at 25°C. Harvested animals were fixed in 1 mL acetone at -20°C overnight (ON), washed with PBS + 0.1% Tween-20 (PBS-T), and stained with MicroB FISH probe against *N*. *parisii* rRNA as previously described [1, 9]. Stained samples were blinded and mounted on glass slides in Vectashield with DAPI (Vector Laboratories). FISH stained parasite sporoplasms, an early, single nucleated stage of the parasite, were counted in 16–24 animals per sample using a Zeiss AxioImager microscope with a 10X objective.

The spore production assay was performed as described with modifications [22]. Synchronized *eri-1* L1s were plated on 6 cm RNAi plates with RNAi bacteria in triplicate and grown 24 hours at 20°C, then transferred to fresh RNAi culture plates containing 2.0 x 10^6^ spores and infected at 25°C for 40 hours, with a transfer to a fresh RNAi plate at 24 hpi to prevent starvation. Animals were fixed in 1 mL acetone at -20°C ON, washed with PBS-T, and loaded onto the COPAS Biosort (Union Biometrica) to dispense 50 animals per well of a 96-well plate. Animals in each well were lysed and stained ON in 100 μL PBS with 1% sodium dodecyl sulfate (SDS), 1% 2-mercaptoethanol (2ME), 1 mg/mL direct yellow 96. The number of spores was counted twice per sample on a hemocytometer with the AxioImager microscope at 10X. After COPAS Biosort dispensing of animals into wells, each well was spot-counted to verify the number of animals per well, and this number was used in the final calculation of spores produced per animal.

### Generation of transgenic *C*. *elegans* strains

The *F56A8*.*3* promoter-mCherry fusion strains ERT173 and ERT174 were made by Gateway cloning (Life Technologies). Briefly, 1755 nucleotides of genomic DNA upstream of the *F56A8*.*3* start codon was amplified by PCR and cloned into the pDONR P4-P1r vector. This DNA was then recombined into the destination vector pSS-5 generously provided by Dr. Supriya Srinivasan, containing in frame mCherry with the *unc-54* 3' UTR (plasmid pET336). This promoter-mCherry fusion was co-injected with the dominant marker *rol-6* to allow selection of transgenic animals with a roller (*rol*) phenotype. Three independent lines were verified for similar mCherry expression. Two of these transgenic strains are ERT173 *jyEx77[pF56A8*.*3a*::*mCherry*::*unc54 3’UTR]* and ERT174 *jyEx78[pF56A8*.*3a*::*mCherry*::*unc54 3’UTR]*

### Antibody production and immunological techniques

A recombinant F56A8.3 protein consisting of the entire N-terminal domain (F56A8.3-NT) (AA 1–243) was used for antibody production at ProSci, Inc (Poway, CA). Briefly, the 729 bp of DNA downstream of the start codon was cloned from cDNA by Gateway cloning into the pDONR221 vector and recombined into the destination vector pET-DEST42 with a C-terminal V5 and 6xHis tags. F56A8.3 protein was induced and purified from *E*. *coli* BL21(DE3) pr1952 [23], with Ni-NTA matrix (Qiagen) as described [24]. Two rabbits were immunized with 200 mg of purified F56A8.3-NT in complete Freund's adjuvant (Sigma) at week 0 and boosted with 100 mg of antigen in incomplete Freund's adjuvant (Sigma) at weeks 2 and 4 and thereafter every 4 weeks by ProSci Inc. The resulting sera, collected 1 week after each boost, were pooled, and the IgG was purified with protein A-agarose (Invitrogen).

For immunohistochemistry, N2, WU1236, and GH351 animals were anesthetized with 10 mM levamisole, their intestines dissected out, and fixed for 1 hour in 4% paraformaldehyde (PFA). For N2, dissected intestines were stained with anti-F56A8.3-NT diluted to 10 mg/mL in block buffer (PBS, 0.5% Triton X-100, 1 mM EDTA, 5% BSA, 0.05% NaN_3_) for 1 hour, followed by Cy3-conjugated goat anti-rabbit (Jackson Immunoresearch) at 2 mg/mL in block. For WU1236 and GH351, intestines were stained as above with anti-F56A8.3-NT, together with the mouse primary antibody anti-GFP (Roche) at 10 mg/mL and the secondary antibody FITC-conjugated goat anti-mouse (Life Technologies) at 2 mg/mL.

### CRISPR-Cas9 Targeted Genome Editing

For CRISPR disruption of *F56A8*.*3*, we used the technique previously described using the plasmids generously donated by Dr. John Calarco, *Peft-3*::cas9-SV40_NLS::*tbb-2* 3'UTR (Addgene plasmid 46168) and PU6::*klp-12*-sgRNA (Addgene plasmid 46170) [25]. Briefly, an sgRNA targeting the 5' end of *F56A8*.*3* was created using round-the-horn cloning for site directed mutagenesis of PU6::*klp-12*-sgRNA, replacing the *klp-12* sgRNA sequence with the *F56A8*.*3* sequence ATCGCATAAATATAGTCTGA [26]. N2 animals were injected with 112.5 ng/μL total DNA, with *Peft-3*::cas9 at 50 ng/μL, *F56A8*.*3* sgRNA at 45 ng/μL, and the dominant mCherry markers pGH8 at 10 ng/μL, pCFJ104 at 5 ng/μL, and pCFJ90 at 2.5 ng/μL. Transgenic F1 progeny were screened for mCherry expression and plated individually. After F2 progeny production, F1 animals were screened by single animal PCR using primers to amplify an 80 bp product spanning the Cas9 cut site. PCR products were run on 10% TBE gels (BioRad) to identify heterozygote mutants that had a wild-type 80 base pair (bp) PCR product, together with a larger or smaller PCR product, presumably generated by non-homologous repair after Cas9 cleavage. From these experiments we generated strain ERT325 *F56A8*.*3(jy4)*.

For CRISPR-mediated deletion of the *F56A8*.*3* region, a homologous recombination (HR) donor template was PCR cloned from genomic DNA to contain the 1796 bp upstream and 1790 bp downstream of the *F56A8*.*3* start and stop codons, respectively. These recombination arms were cloned into the Gateway donor vectors pDONR P4-P1r and pDONR P2r-P3, respectively, and the gene *Prps-0*::*HygR*::*gpd-2*::*GFP*::*unc-54 3'UTR* was cloned from the IR99 plasmid (generously donated by Dr. Jason Chin) into pDONR221, flanked by LoxP sites [27]. These three vectors were recombined into the destination vector pDEST R4-R3. N2 animals were injected with a 200 ng/μL total DNA mix, with *Peft-3*::cas9 at 50 ng/μL, *F56A8*.*3* sgRNA at 50 ng/μL, *F56A8*.*3* HR donor template at 50 ng/μL, and pRF4 (*rol-6)* at 50 ng/μL. Transgenic F1 progeny were screened by GFP and *rol-6* expression and plated individually. After F2 progeny production, plates were screened for 75% segregation of GFP among the F2s to indicate an HR event. A *F56A8*.*3* knockout was verified by PCR for loss of *F56A8*.*3* with internal primers, and for gain of the *Prps-0*::*HygR*::*gpd-2*::*GFP*::*unc-54 3'UTR* into the *F56A8*.*3* locus by junction PCR. From these experiments we generated the strain ERT424 *F56A8*.*3(jy8)*.

## Results

### An RNAi screen identifies genes important for *C*. *elegans* larval arrest induced by *N*. *parisii* infection

**Fig 1:**
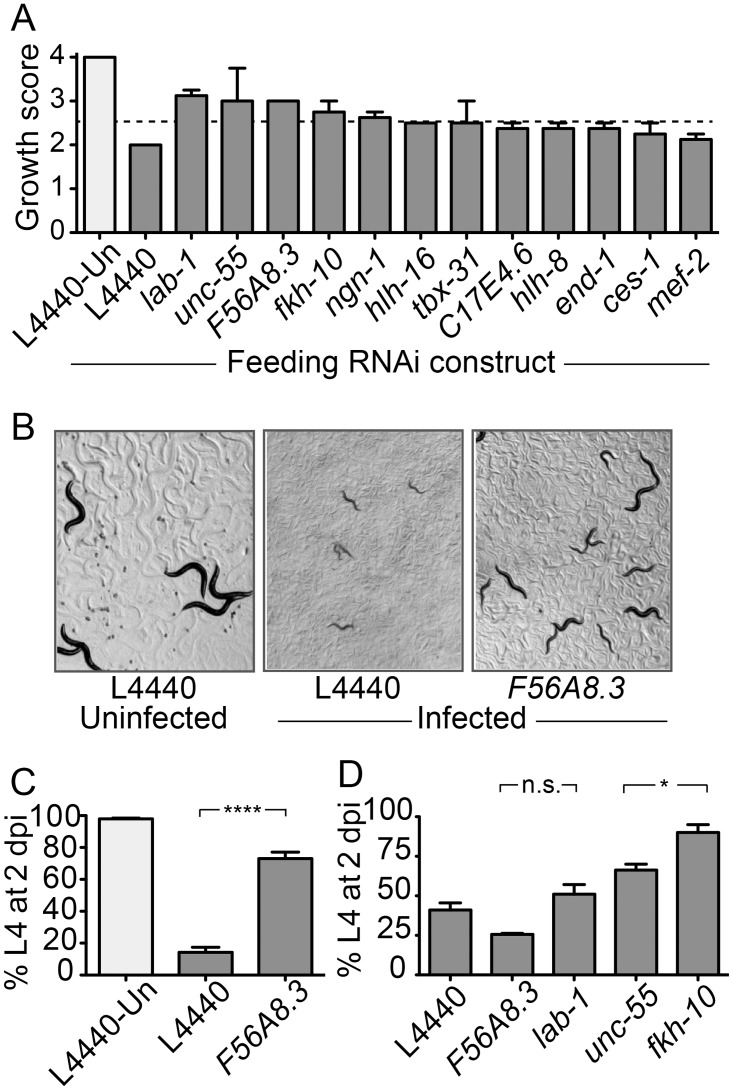
RNAi screen for host genes that regulate *N*. *parisii-*induced larval arrest of *C*. *elegans*. (A) Twelve RNAi clones that were originally scored as hits in the screen were retested, using a semi-quantitative visual score of *C*. *elegans eri-1* strain growth on feeding RNAi at 2 days post-infection (dpi). Uninfected animals grown on L4440 (control RNAi, light gray) provided the baseline for fully developed worms and were scored as a four, while fully arrested worms were scored as a one. Re-tested RNAi clones were scored under infected conditions (dark gray). Twelve hits are shown, consisting of 10 transcription factors, one LRR gene (*F56A8*.*3*), and one contaminant (gene identity different from what was listed in the library) belonging to neither gene class (*lab-1*). Data are represented as mean values with standard error of the mean (SEM) from biological duplicates in two independent screens. (B) Representative images of larval arrest upon *N*. *parisii* infection, and inhibition of arrest after *F56A8*.*3* RNAi. (C) Larval arrest on control or *F56A8*.*3* RNAi after *N*. *parisii* infection (dark gray) measured as the percent of animals reaching the L4 larval stage at 2 dpi. Uninfected animals grown on L4440 (control RNAi, light gray) are shown for comparison. Data are represented as mean values with SEM from seven independent experiments (****p<0.0001, unpaired two-tailed t-test). (D) Larval arrest on feeding RNAi after *P*. *aeruginosa* infection, quantified as the percent of animals reaching the L4 larval stage at 2 dpi. Data are representative of two independent experiments, with the mean and SEM from biological duplicates shown (n.s. is not significant and * is p<0.05 compared to L4440 in one-way analysis of variance with Dunnett’s multiple comparison test).

Previously, we described that *N*. *parisii* is a natural microsporidian pathogen of *C*. *elegans* that causes a lethal intestinal infection [9]. Here we report the finding that infection of first larval stage (L1) *C*. *elegans* animals with very high doses of *N*. *parisii* spores leads to an arrest in larval development (see Fig 1A–1C). By contrast, L1s infected with a high dose of killed, non-infectious spores develop normally, indicating that larval arrest after *N*. *parisii* exposure is a host response to an active *N*. *parisii* infection, and not simply a response to the presence of a high does of *N*. *parisii* spores (data not shown). We used the easily scored, visual phenotype of larval arrest to conduct an RNAi screen for host genes required for *N*. *parisii* infection/development, using libraries of predicted transcription factors and LRR proteins. The initial screen included approximately 436 genes, and 12 genes were initially scored as hits. We repeated the larval arrest assay with these twelve hits and found that five of them consistently blocked larval arrest after infection, with a visual score of greater than 2.5 (Fig 1A). An interesting hit from this screen was the RNAi clone against a gene coding for a predicted transmembrane LRR protein *F56A8*.*3* (Gene ID: 176740). There is conservation of the LRR-containing, N-terminal region of F56A8.3 with a protein found in numerous animal species, including leucine-rich repeat-containing protein 59 (LRRC59) in humans. RNAi against this gene in *C*. *elegans* caused a significant inhibition of arrest at 2 days post-infection (dpi) when compared to the L4440 RNAi control (Fig 1B). These results were consistent across seven independent experiments with approximately 70% of infected *F56A8*.*3* RNAi-treated animals reaching the L4 stage at 2 dpi, compared to approximately 13% of infected control RNAi animals (Fig 1C). Under these conditions almost 100% of uninfected wild-type animals reached the L4 stage. The sequence for the *F56A8*.*3* RNAi clone is provided (S1 Text).

A defect in *C*. *elegans* larval development occurs after exposure to a number of pathogens, including the Gram-negative bacterial pathogen *Pseudomonas aeruginosa* [28]. In order to test for the specificity of this effect, four of the strongest RNAi hits from the *N*. *parisii* screen were tested for their effect on larval development after infection with *P*. *aeruginosa*. We found that RNAi of some genes, like *unc-55* and *fkh-10*, also inhibited arrest on *P*. *aeruginosa*, suggesting they are important for a general larval arrest response, or slowing of development upon infection (Fig 1D) [29, 30]. However, RNAi of *F56A8*.*3* failed to prevent larval arrest on *P*. *aeruginosa*, suggesting specificity of this RNAi clone to *N*. *parisii* infection (Fig 1D). Therefore, we chose to focus on *F56A8*.*3* because of this strong, consistent larval arrest inhibition by the *F56A8*.*3* RNAi clone after *N*. *parisii* infection but not after *P*. *aeruginosa* infection.

### RNAi against *F56A8*.*3* results in lower *N*. *parisii* pathogen load

**Fig 2:**
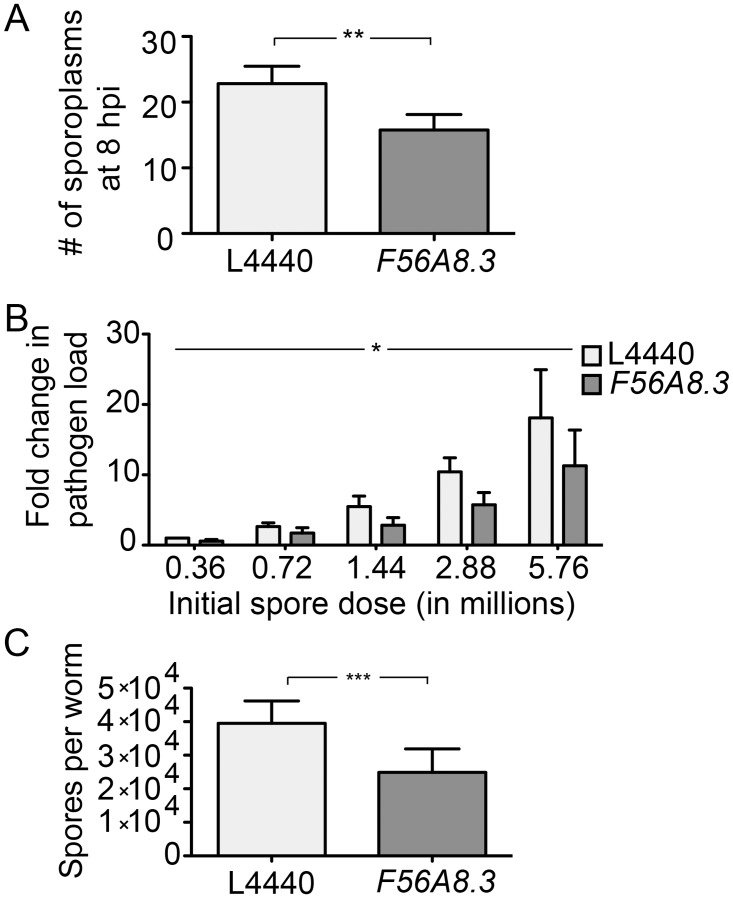
*F56A8*.*3* RNAi clone reduces the level of *N*. *parisii* infection at several stages of infection. (A) Pathogen load at 8 hpi on control or *F56A8*.*3* RNAi measured as the number of FISH-stained sporoplasms seen in intact *C*. *elegans* intestines. Data are represented as mean values with SEM from three independent, blinded experiments (**p = 0.002, paired two-tailed t-test). (B) Pathogen load at 30 hpi on control or *F56A8*.*3* RNAi measured as the fold change in *N*. *parisii* rDNA transcript by qRT-PCR relative to L4440 infected at the lowest dose. Data are represented as mean values with SEM from three independent experiments (*p = 0.033, two-way analysis of variation, testing RNAi treatment effecting pathogen load at all doses). (C) Pathogen load at 40 hpi with *C*. *elegans* infected at the L2 stage on control or *F56A8*.*3* RNAi measured as the average number of spores produced per animal. Data are represented as mean values with SEM from three independent experiments (***p = 0.0005, paired two-tailed t-test).

The lack of larval arrest seen on *F56A8*.*3* RNAi could be due to lower pathogen load or due to disruption of the pathways that mediate arrest upon *N*. *parisii* infection. To test if the *F56A8*.*3* RNAi clone affected pathogen load, we performed a series of experiments to quantify the *N*. *parisii* parasites at various time points after infection, which represent different stages of the parasite life cycle. *N*. *parisii* appears to have a life cycle similar to most microsporidia. Briefly, microsporidian spores fire an infection apparatus called a polar tube to inject their contents directly into host cells, where they replicate and ultimately differentiate back into infectious spores that exit hosts to infect other individuals [4, 9]. At 8 hours post infection (hpi) in *C*. *elegans*, we can use FISH-staining to visualize the early, single nucleated stage of the parasite called a 'sporoplasm' [1]. Here, we found that there were about 40% fewer *N*. *parisii* sporoplasms after *F56A8*.*3* RNAi compared to empty RNAi vector controls when measured by FISH against *N*. *parisii* rRNA (Fig 2A). At 30 hpi, *N*. *parisii* has replicated as multi-nucleate meronts throughout the intestine, but mature spores are not yet visible [1, 12]. Here, we found an approximately 50% reduction in pathogen load after *F56A8*.*3* RNAi across five different spore doses as measured by qRT-PCR for *N*. *parisii* rRNA (Fig 2B). Similar results were seen with qPCR for *N*. *parisii* β-tubulin transcript at the same doses and time point (S1 Fig). At 40 hpi, when the parasitic meronts have differentiated back into spores, we found that *F56A8*.*3* RNAi resulted in approximately 40% reduction in the number of spores produced per animal compared to RNAi controls (Fig 2C). The experiments described above were performed with *C*. *elegans* infected after the L1 stage (see Materials and Methods), while the larval arrest experiments infected *C*. *elegans* at the L1 stage. Indeed, similar results were seen when the animals were infected at the L1 stages, with *F56A8*.*3* RNAi resulting in lower spore load (S2 Fig). Together, these results show that RNAi against *F56A8*.*3* results in lower *N*. *parisii* pathogen load across the majority of stages in the parasite life cycle. This lesser pathogen burden could explain the inhibition of larval arrest caused by this RNAi clone, as we show that increasing *N*. *parisii* pathogen load results in decreasing worm size (S3 Fig). We concluded from these results that the *F56A8*.*3* RNAi clone was reducing the expression of a protein required for *N*. *parisii* infection.

### *F56A8*.*3* protein is found on lysosome related organelles (LROs) in the *C*. *elegans* intestine

**Fig 3:**
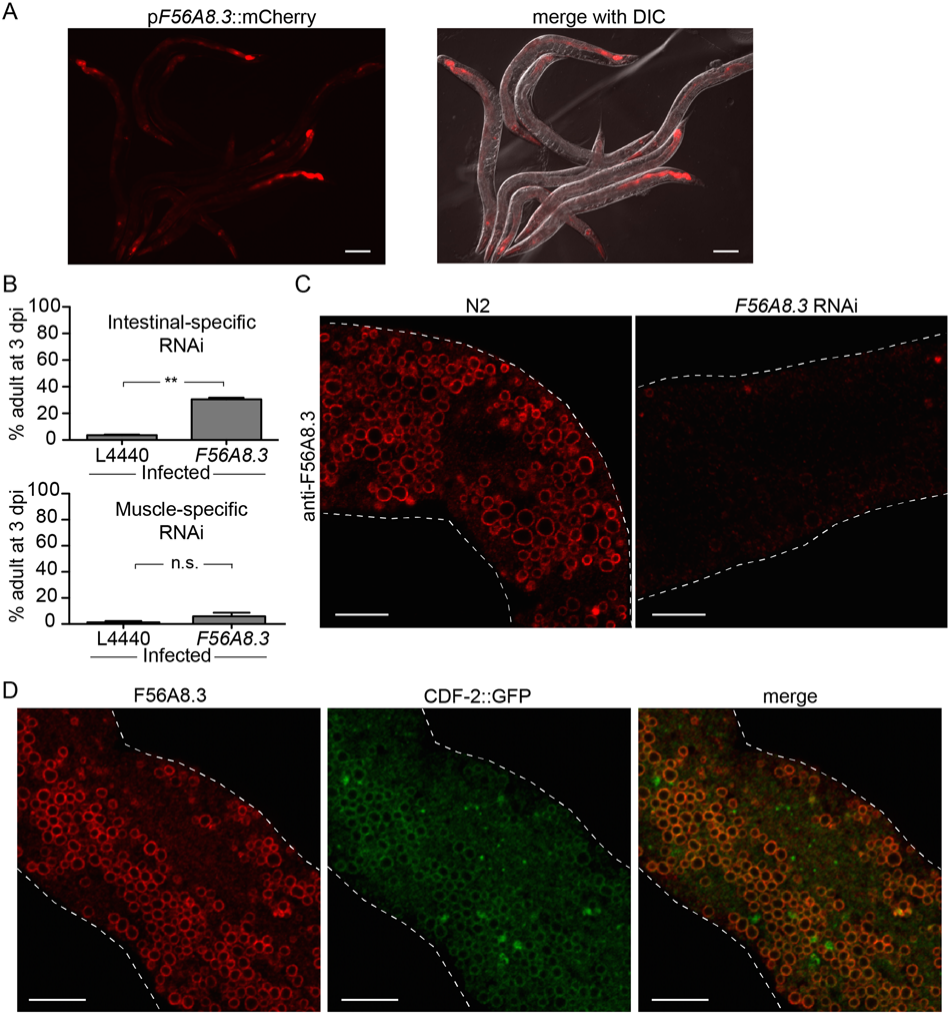
*F56A8*.*3* RNAi clone acts in the intestine, and the F56A8.3 protein localizes to lysosome-related organelles in the intestine. (A) Representative image of transgenic *C*. *elegans* expressing intestinal mCherry under control of the putative *F56A8*.*3* promoter. Scale bar = 100 μm. (B) Larval arrest of tissue-specific RNAi strains MGH167 (left, intestinal-specific) and SPC272 (right, muscle-specific) after *N*. *parisii* infection, measured as the percent animals reaching the adult stage at 3 dpi. Data are represented as mean values with SEM from two independent experiments (**p = 0.002; n.s. p = 0.26, unpaired two-tailed t-test). (C) Representative image of endogenous F56A8.3 localization in dissected intestines from wild-type N2 *C*. *elegans* (left) or from N2 treated with *F56A8*.*3* RNAi (right). F56A8.3 was detected with anti-F56A8.3 followed by goat anti-rabbit IgG conjugated to Cy3. Scale bar = 10 μm. (D) Representative image of endogenous F56A8.3 colocalization relative to CDF-2::GFP in the WU1236 transgenic strain. F56A8.3 was detected as in C; CDF-2 GFP was detected with anti-GFP followed by anti-mouse IgG conjugated to FITC. Scale bar = 10 μm.

Before we learned that the *F56A8*.*3* RNAi clone was likely acting off-target to affect *N*. *parisii* infection (see results below), we studied the *F56A8*.*3* target gene further. *F56A8*.*3* encodes a single-pass transmembrane protein with five leucine-rich repeats (LRRs). Since LRR domains are found in numerous proteins that play a role in host-pathogen interactions, such as Toll-like and NOD-like receptors (TLRs and NLRs, respectively) [31, 32], we sought to further characterize the role of *F56A8*.*3* in *N*. *parisii* infection. First, we investigated the tissue expression of *F56A8*.*3* by creating a transgenic strain that expressed mCherry under control of the putative *F56A8*.*3* promoter. This promoter drove mCherry expression in the intestine, pharynx, and some anterior and posterior neurons (Fig 3A). Given that *N*. *parisii* infection occurs exclusively in the intestine of *C*. *elegans*, we conducted tissue-specific RNAi to investigate if *F56A8*.*3* was acting in the intestine to facilitate infection. Using the intestinal-specific RNAi strain MGH167, we found that *F56A8*.*3* RNAi resulted in significant inhibition of larval arrest compared to the RNAi control (Fig 3B). By contrast, there was no significant effect of this RNAi clone on larval arrest in the muscle-specific RNAi strain SPC272. These results suggest that the *F56A8*.*3* RNAi clone acts in the intestine to inhibit *N*. *parisii* infection.

We next investigated the subcellular localization of the endogenous F56A8.3 protein in *C*. *elegans* intestinal cells. We generated rabbit anti-F56A8.3 polyclonal antibodies using an N-terminal domain of F56A8.3 (from the initial methionine to the beginning of the transmembrane domain) expressed and purified from *E*. *coli*. This antibody recognized a protein band of the correct size on Western blots (see below). With immunohistochemistry of dissected *C*. *elegans* intestines, we found that anti-F56A8.3 localized to the membrane surrounding large circular structures in the intestine generically called “gut granules” (Fig 3C, left) [33]. This antibody signal was noticeably reduced in wild type animals treated with *F56A8*.*3* RNAi (Fig 3C, right), further confirming the specificity of the antibody and indicating that this RNAi clone does indeed reduce expression of endogenous *F56A8*.*3* protein. In addition, we found that F56A8.3 colocalized with the zinc transporter CDF-2, which is found on a subset of gut granules called lysosome-related organelles (LROs) (Fig 3D) [17]. Similarly, F56A8.3 colocalized with another LRO membrane marker in the intestine, GLO-3 (S4 Fig) [16].

### Mutation of the *F56A8*.*3* gene does not recapitulate the phenotype of the RNAi clone

**Fig 4:**
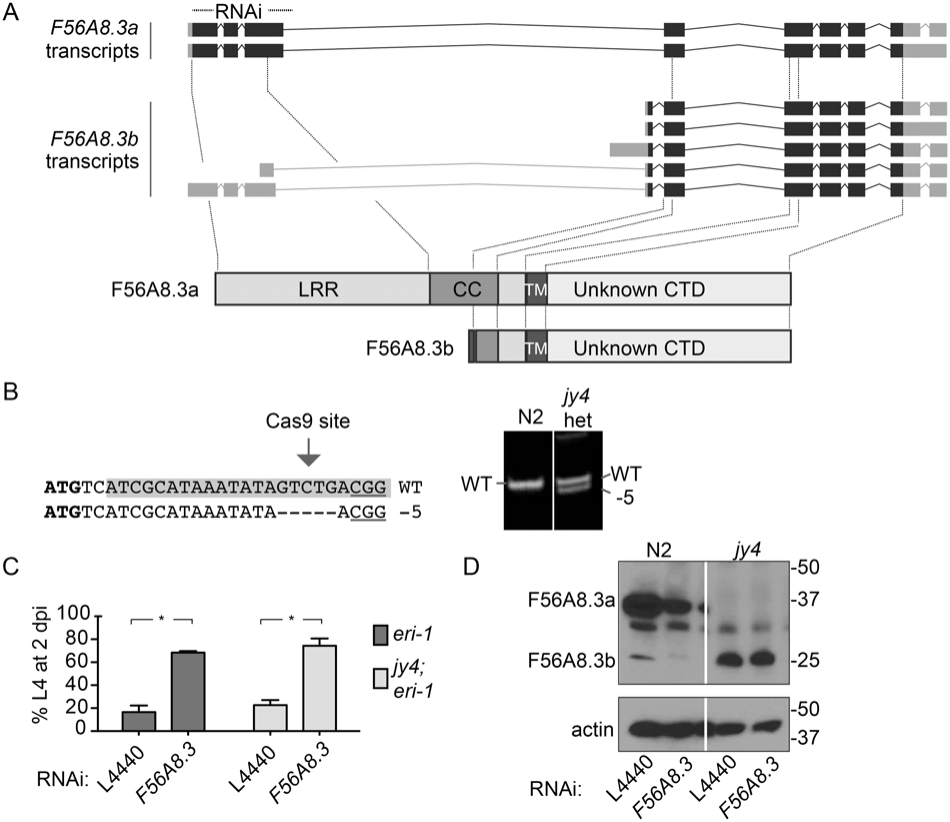
Mutation of *F56A8*.*3a* does not recapitulate the larval arrest phenotype of *F56A8*.*3* RNAi. (A) *Top*: Schematic representation of the *F56A8*.*3a* and *F56A8*.*3b* pre-mRNA transcripts, with exons represented as black and gray blocks, indicated coding and non-coding sequences, respectively, and solid lines representing introns. The sequence covered by *F56A8*.*3* RNAi clone is indicated at the top as a dotted line. Image adapted from WormBase and based on EST data (WBGene00010139). *Bottom*: Schematic representation of F56A8.3a and F56A8.3b protein, with dotted lines showing the relative locations on the coding exons from which the main protein domains are derived (LRR is the leucine-rich repeat domain, CC is the coiled coil domain, TM is the transmembrane domain, and CTD is the C-terminal domain). (B) Representation of CRISPR-Cas9 genome editing of the 5'-most exon of the *F56A8*.*3* gene, with the *F56A8*.*3* start codon in bold, the sgRNA targeting sequence highlighted, and the protospacer adjacent motif (PAM) underlined (left). WT is the wild-type sequence found in N2, and -5 is a 5 bp deletion found in the mutant ERT327 (*jy4*), with representative 80 bp and 75 bp PCR products from the F1 screen shown (right). (C) Larval arrest of *eri-1* and *F56A8*.*3* frameshift mutant ERT360 *F56A8*.*3(jy4)* (in an *eri-1* background) on control or *F56A8*.*3* RNAi after *N*. *parisii* infection, measured as the percent animals reaching the L4 at 2 dpi. Data are represented as mean values with SEM from two independent experiments (*p = 0.013 (left), *p = 0.022 (right), unpaired two-tailed t-test). (D) F56A8.3 protein in N2 and *F56A8*.*3* frameshift mutation ERT327 *F56A8*.*3(jy4)* on either control (L4440) or *F56A8*.*3* RNAi. The top picture represents a single blot probed with anti-F56A8.3 antibodies, while the bottom represents a single blot probed with anti-actin. Indicated molecular weight markers are in kilodaltons (kD).

The *N*. *parisii* infection phenotypes we saw with the *F56A8*.*3* gene were all conducted using RNAi. We confirmed that the *F56A8*.*3* transcript was knocked down by the RNAi clone (S5 Fig), and that levels of the endogenous protein were reduced (Fig 3C). However, we wanted to confirm that inhibition of larval arrest and reduced pathogen load from the RNAi clone was due to this *F56A8*.*3* knockdown and not due to an off-target effect. Since there were no existing null mutants for *F56A8*.*3*, we created a null mutant using the CRISPR-Cas9 system for targeted genome editing. The *F56A8*.*3* genomic region is predicted to encode two separate proteins, a full length protein called isoform a (F56A8.3a), and a truncated protein called isoform b (F56A8.3b), which lacks approximately 79% of the N-terminal region upstream of the TM domain, including the entire LRR domain (Fig 4A, bottom). The *F56A8*.*3a* transcripts encompass the entire coding region, while the *F56A8*.*3b* transcripts vary dramatically in length, with some containing only the downstream coding regions and other covering the entire coding region (Fig 4A, top). The *F56A8*.*3* RNAi clone targets the 5’ coding region of the *F56A8*.*3* (indicated with a dotted line at the top of Fig 4A), which would be predicted to knockdown all transcript isoforms from the *F56A8*.*3a* gene. Therefore, we created an *F56A8*.*3a* mutant by designing a single-guide RNA (sgRNA) targeting the 5’ coding region of *F56A8*.*3a* gene (Fig 4B). Using the protocol developed by Friedland *et al*. [25], we isolated a 5 bp deletion in the early *F56A8*.*3* coding region, which is predicted to cause a frameshift and early stop codon (Fig 4B). This frameshift mutant *F56A8*.*3 (jy4)*, showed a complete loss of *F56A8*.*3a* protein as detected by Western blot (Fig 4D, right). However, when the *F56A8*.*3(jy4)* mutant was infected with *N*. *parisii*, we saw no inhibition of larval arrest compared to the control (Fig 4C). Furthermore, *F56A8*.*3* RNAi treatment of *F56A8*.*3(jy4)* mutants caused an inhibition of arrest similar to control animals on *F56A8*.*3* RNAi (Fig 4C). These experiments were performed in an *eri-1* mutant background, because *eri-1* mutants exhibited more robust larval arrest than wild-type N2 animals. Altogether, these data suggest that the larval arrest and pathogen load phenotypes seen on *F56A8*.*3* RNAi are not due to knockdown of F56A8.3a protein.

### A complete deletion of the *F56A8*.*3* genomic region does not recapitulate the infection phenotypes seen with *F56A8*.*3* RNAi

**Fig 5:**
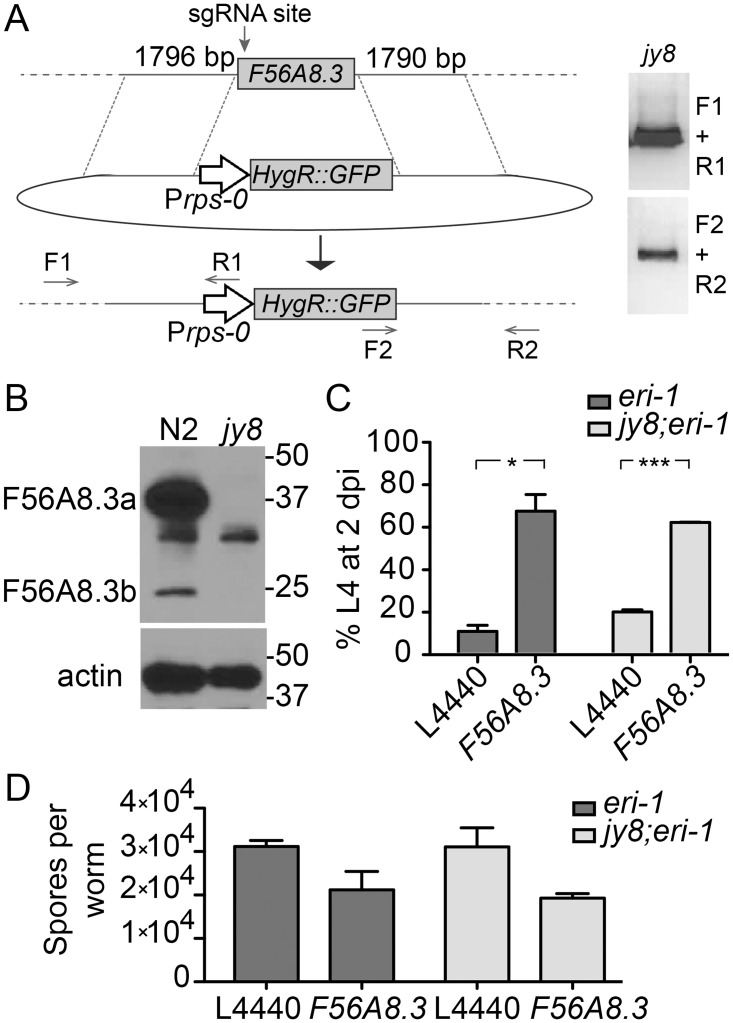
A complete deletion of the *F56A8*.*3* locus does not recapitulate the larval arrest phenotype of *F56A8*.*3* RNAi. (A) Schematic representation of donor homologous repair template and predicted recombinant product after CRISPR-Cas9 mediated cutting of *F56A8*.*3* (at the indicated sgRNA site). The donor template (middle) contains homologous regions of 1796 bp upstream and 1790 bp downstream of the *F56A8*.*3* start and stop codons, respectively, which flank a P*rps-0*-controlled hygromycin-resistance gene (HygR) with an intergenic GFP expressed as part of an operon. Primer pairs F1+R1 and F2+R2 (bottom) flank the upstream and downstream genomic insertion sites, and their PCR products were detected in knockout mutant ERT425 *F56A8*.*3(jy8)* (right). (B) F56A8.3 protein in N2 and *F56A8*.*3* knockout mutation ERT425 *F56A8*.*3(jy8)* detected with anti-F56A8.3. Indicated molecular weight markers are in kilodaltons (kD), and actin loading controls are shown (bottom). (C) Larval arrest of *eri-1* and ERT430 *F56A8*.*3(jy8)* knockout (in an *eri* background) on control or *F56A8*.*3* RNAi after *N*. *parisii* infection, measured as the percent animals reaching the L4 stage at 2 dpi. Data are represented as mean values with SEM from two independent experiments (*p = 0.021 (left), ***p = 0.0006 (right), unpaired two-tailed t-test). (D) Pathogen load at 40 hpi of *eri-1* and ERT430 *F56A8*.*3(jy8)* knockout (in an *eri-1* background) on control or *F56A8*.*3* RNAi measured as the number of spores produced per animal. Data are represented as mean values with SEM from three biological replicates.

We next sought to determine whether the truncated F56A8.3b protein was playing a role in *N*. *parisii* infection, because there are some *F56A8*.*3b* transcripts that could be targeted by the *F56A8*.*3* RNAi clone (Fig 4A). In support of this idea, we found that wild-type animals treated with *F56A8*.*3* RNAi showed a reduction in F56A8.3b protein, as well as F56A8.3a protein, although the decrease of F56A8.3b protein was not noticeable in *F56A8*.*3(jy4)* animals (Fig 4D). In fact, the amount of F56A8.3b protein appears to increase in the F56A8.3a null mutant *F56A8*.*3(jy4)* compared to N2 wild-type animals (Fig 4D, comparing left panel to right panel) which may represent a compensatory mechanism by the mutant. Thus, it was possible that the several-fold increase in F58A8.3b protein seen in *F56A8*.*3(jy4)* was rescuing these animals to WT infection levels. To determine if *F56A8*.*3b* plays a role in *N*. *parisii* infection, we knocked out the entire *F56A8*.*3* locus and replaced it with a GFP transgene. This editing was done using the same CRISPR-Cas9 technique described earlier, but included a donor vector containing a GFP transgene driven flanked by 1796 bp and 1790 bp upstream and downstream of the *F56A8*.*3* coding region, respectively (Fig 5A). This donor vector was designed to completely remove the *F56A8*.*3* gene, which includes the entire sequence homologous to the *F56A8*.*3* RNAi clone. We screened for successfully engineered genomes via GFP expression in the F1 progeny of the injected animals, and verified with PCR to span the upstream and downstream insertion sites (Fig 5A, *right*). We isolated and homozygosed one knockout *F56A8*.*3* mutant *F56A8*.*3(jy8)*. This mutant completely lacked both F56A8.3a and F56A8.3b protein, as detected by Western blot analysis (Fig 5B). However, the *F56A8*.*3(jy8)* mutants exhibited no inhibition of larval arrest compared to the control animals after infection with *N*. *parisii* (Fig 5C), and treatment of *F56A8*.*3(jy8)* animals with *F56A8*.*3* RNAi showed an inhibition of arrest similar to control animals on *F56A8*.*3* RNAi. We also analyzed spore production and found that *F56A8*.*3(jy8)* mutants had no difference in spore production at 40 hpi when compared to control animals, yet showed a similar reduction after *F56A8*.*3* RNAi (Fig 5D). Altogether, these results indicate that the infection phenotypes seen on the *F56A8*.*3* RNAi clone are likely due to an off-target RNAi effect of the *F56A8*.*3* RNAi clone.

## Discussion

Here, we report *N*. *parisii*-induced larval arrest in *C*. *elegans* and the results of an RNAi screen to identify host genes that conferred susceptibility to microsporidian infection. We chose to focus on the *F56A8*.*3* because its RNAi clone was one of the strongest and most robust hits in this screen, and because this gene encodes a conserved transmembrane protein containing an LRR domain, which we hypothesized was being hijacked by *N*. *parisii* for infection. While we were able to characterize an effect of *F56A8*.*3* RNAi on infection and show that the F56A8.3 target is knocked-down by the RNAi clone, we ultimately found that the *F56A8*.*3* gene is not responsible for the larval arrest phenotype. These findings provide a cautionary tale about relying solely on an RNAi clone to assess the functional relevance of a gene. However, our description of larval arrest upon infection, results from an RNAi screen for host genes important for microsporidian infection, and characterization of the expression and localization of the F56A8.3 protein provide information and tools useful for further study of this phenotype and protein.

### Microsporidian infection induces larval arrest in *C*. *elegans*

Larval arrest is among a range of phenotypes induced by microsporidian infection. These phenotypes can vary from benign to lethal depending on the species of pathogen and host involved. Acute microsporidian infection places a substantial burden on the resources of the host, as all of the pathogen replication occurs inside host cells. Thus, it is likely that *C*. *elegans* larvae can sense (e.g. through a nutritional cue) the extensive *N*. *parisii* replication occurring in their intestinal cells and then arrest development [1]. Other microsporidian infections have been described to arrest development of their hosts, such as *Nosema whitei* infection of the flour beetle [34, 35], and *Nosema bombycis* infection of the silkworm [36]. We used this microsporidia-induced larval arrest to conduct an RNAi screen for host genes that mediate susceptibility to *N*. *parisii* infection. Indeed, one of the strongest hits in this screen (*F56A8*.*3* RNAi) resulted in lower pathogen load at multiple parasite stages. Thus, if larval arrest is a result of substantial pathogen burden on the host, then inhibition of pathogen growth via this RNAi clone would be expected to relieve some of this burden and allow larval development. However, it is possible that the *F56A8*.*3* RNAi clone is also able to disrupt the pathways that mediate arrest upon *N*. *parisii* infection.

### *C*. *elegans* RNAi screen for host genes important for microsporidian infection identifies the *F56A8*.*3* RNAi clone

The *F56A8*.*3* RNAi clone was a hit from our screen and this RNAi clone was capable of knocking down the *F56A8*.*3* transcript and its protein products. However, the *N*. *parisii* infection phenotypes were not due to knockdown of this gene, based on our analysis of two *F56A8*.*3* mutants we made, including a complete deletion mutant. It is likely that the *F56A8*.*3* RNAi clone is knocking down another *C*. *elegans* gene, although candidate genes are not obvious based on homology to the *F56A8*.*3* RNAi clone sequence. In fact, only two other *C*. *elegans* genes were identified as weak putative targets of the RNAi clone using the program dsCheck, *fcd-2* and *F57E7*.*1*, and neither of these genes transcripts were reduced on *F56A8*.*3* RNAi, as measured by qRT-PCR (data not shown) [37]. It is likely that this off-target host gene is acting to facilitate *N*. *parisii* infection progression in the intestines of *C*. *elegans*, based on the reduced pathogenic outcome at numerous time points of infection, and the specificity of the larval arrest phenotype when RNAi was restricted to the intestine. It is also a possibility that the *F56A8*.*3* RNAi clone is knocking down a microsporidian transcript needed for pathogen replication. However, this would require pathogen transcripts to be secreted from an early, intracellular parasitic stage into the intestinal cytoplasm, or require the pathogen to take up small RNAs derived from the *F56A8*.*3* RNAi clone to have a biological impact in the parasite. Although some microsporidian genomes encode for RNAi machinery, *N*. *parisii* is not one of those genomes, suggesting it does not undergo RNAi [1]. Furthermore, BLAST analysis of the *N*. *parisii* ERTm1 genome did not identify any regions of the genome that would be targeted by this RNAi clone (data not shown). Thus, the mechanism by which the *F56A8*.*3* RNAi clone blocks infection-induced larval arrest remains unresolved.

### The *F56A8*.*3* gene encodes a predicted transmembrane domain protein with LRR repeats that localizes to intestinal lysosome-related organelles

Because the *F56A8*.*3* gene initially appeared to be responsible for the infection-induced phenotype, we characterized the function and expression of this gene, and developed useful reagents for its future study. *F56A8*.*3* encodes a predicted single-pass transmembrane protein with an N-terminal LRR and coiled-coil domain, and a large C-terminal domain of unknown function (Fig 4A). The N-terminal domain, which includes the entire region upstream of the transmembrane domain, is conserved across numerous animal species including humans. In *C*. *elegans*, we found that the 5’ region directly upstream of the *F56A8*.*3* start codon acts to drive transgene expression in the intestine, pharynx, and some neurons. Furthermore, we found that the endogenous F56A8.3 protein localized in the intestine to LROs, acidic organelles containing some lysosomal proteins that are thought to perform cell type-specific storage and secretion functions [38]. Some examples of LROs in other animals are melanosomes, platelet-dense granules, and acrosomes found in melanocytes, platelets, and sperm cells, respectively [39, 40]. Currently, only a handful of proteins have been found to localize to LROs in the *C*. *elegans* intestine, including CDF-2 and GLO-1. Despite comprising a significant portion of the intestine, the function of these organelles is still relatively unknown, aside from their role in zinc storage to limit zinc toxicity [17, 41, 42].

In *C*. *elegans*, the *F56A8*.*3* gene was predicted to encode for two protein products, the full-length F56A8.3a and a truncated F56A8.3b that lacks approximately 75% of the N-terminal domain, including the entire LRR domain (Fig 4A). We show with Westerns blots that both of these proteins are indeed expressed in *C*. *elegans*, and that the *F56A8*.*3* RNAi clone can knockdown both proteins. We generated two mutants in these proteins using the CRISPR-Cas9 system: *F56A8*.*3a(jy4)* mutants completely lack the *F56A8*.*3a* isoform and *F56A8*.*3(jy8)* mutants completely lack both the *F56A8*.*3a* and *F56A8*.*3b* isoforms. Our analysis showed that in *F56A8*.*3a(jy4)* mutants, the amount of F56A8.3b protein is increased dramatically, which likely represents a compensatory mechanism and suggests that F56A8.3b might be involved in similar functions as F56A8.3a. However, both of these mutants appeared phenotypically normal with no obvious defects. In particular, we observed no defect in the number or appearance of LROs in *F56A8*.*3a(jy4)* mutants compared to wild-type animals (data not shown), so the functions of the F56A8.3 proteins are yet to be determined. The putative human ortholog of *F56A8*.*3* encodes LRRC59. This protein is localized to the ER membrane, with the N-terminal, LRR-containing domain projecting into the cytoplasm where it acts as a receptor for cytoplasmic fibroblast growth factor 1 (FGF1) [43, 44]. In light of the data here, it would be interesting to determine if the F56A8.3a and/or F56A8.3b proteins are acting to regulate the biogenesis or function of LROs in the *C*. *elegans* intestine.

### Future Directions

The promising infection phenotypes seen using the *F56A8*.*3* RNAi clone were ultimately not due to knockdown of *F56A8*.*3*, but likely due to an off-target effect on another *C*. *elegans* gene. In future directions, this off-target gene could be identified by conducting RNA-seq to identify genes with reduced expression in *F56A8*.*3(jy8)* mutants treated with *F56A8*.*3* RNAi, compared to mutants treated with control RNAi. Genes with lowered expression could then be verified for their effects on larval arrest and *N*. *parisii* pathogen load with RNAi **and** mutant analysis. In addition to *F56A8*.*3*, there were several other hits from our screen that could be further explored, with the goal of providing more insight into the host/pathogen interactions that underlie infections by microsporidia.

## Supporting Information

S1 Text DNA sequence for *F56A8*.*3* RNAi clone. (DOCX)

S1 Fig *F56A8*.*3* RNAi clone reduces the level of *N*. *parisii* β-tubulin transcript in animals infected at L2/L3 stage. Pathogen load at 30 hpi on control or *F56A8*.*3* RNAi measured as the fold change in *N*. *parisii* β-tubulin transcript by qRT-PCR relative to L4440 infected at the lowest dose. Animals were infected at L2/L3 stage. Data are represented as mean values with SEM from three independent experiments (**p = 0.0022, two-way analysis of variation, testing RNAi treatment effecting pathogen load at all doses).
(TIF)

S2 Fig *F56A8*.*3* RNAi clone reduces the number of *N*. *parisii* spores produced in *C*. *elegans* infected as L1s. Pathogen load at 40 hpi with *C*. *elegans* infected at the L1 stage on control or *F56A8*.*3* RNAi measured as the average number of spores produced per animal. Data are represented as mean values with SEM from two independent experiments.
(TIF)

S3 Fig *N*. *parisii* pathogen load is inversely correlated with *C*. *elegans* animal size. Animals were plated on L4440 bacteria for 18 hours and then infected for 24 hours using a low (3.63 x 10^5^ spores), medium (1.45 x 10^6^ spores), or high dose (5.80 x 10^6^ spores) of *N*. *parisii* spores on a 10 cm RNAi plate. Pathogen load was measured by FISH to *N*. *parisii* 18s rRNA and the percent area of the animal infected was calculated using ImageJ. Animal size was calculated by ImageJ and presented as the percent of the mean size of uninfected animals conducted in parallel. Data are represented as mean values with SEM of 20 individual animals in a single experiment.
(TIF)

S4 Fig F56A8.3 protein colocalizes with GLO-3::GFP. Representative image of endogenous F56A8.3 colocalization relative to GLO-3::GFP in the GH351 transgenic strain Scale bar = 10 μm.
(TIF)

S5 Fig *F56A8*.*3* RNAi clone reduces the amount of *F56A8*.*3* transcript. qRT-PCR analysis of the amount of *F56A8*.*3* transcript in *C*. *elegans* grown on control or *F56A8*.*3* RNAi measured as the fold change relative to L4440. Transcript levels were normalized to *snb-1*. Data are represented as mean values with SEM from two independent experiments.
(TIF)
