# Supplementary material for: Characterization of Microsporidia-Induced Developmental Arrest and a Transmembrane Leucine-Rich Repeat Protein in Caenorhabditis elegans
Source: PLoS One. 2015 Apr 13;10(4):e0124065. doi: 10.1371/journal.pone.0124065 (PMC4395247; doi:10.1371/journal.pone.0124065)
Supplement: S1 Text — (DOCX) [file pone.0124065.s001.docx]

**S1 Text.** **DNA sequence for *F56A8.3* RNAi clone.**

GAGCTCAAAAACTTGCAAGAAGATGATGAGTTGGATTTATCGGCAAGCGGAATTCAAGAATTCCCCAATGCAATCGTCCAACTGCCACGGTTGACGAAATTGGATCTGAGCTCAAATGCAATCACTTTCTTGCCGGAATCTTTCTGCAAGATGACTAAGCTTATTCGGTAAGAGTTTTAAGCTGAAAATTTTTGTATATTTTAATTTAATTTTTCCCTTTTAAGGTTAGACTTCGGAAGTTGCCAGCTTCATCATCTCCCTGATGGAATCGGGCTCTTGACAAGCTTGCAGCACTTGAACCTTTATAACAATCAAATAGAGGTATTTAAAAAATCGGCTAAGGCATGTAGAAATCAATCAATATTTTCCAGGACTTGCCGCTCTCGTTCGCCAACTTAAAATCCTTGAAGTGGCTGGATTTGAAGAAAAATCCGCTCAACTCGAAGCTCGCCGCCATCGCAGGAAACTGTGGGACTGATGCCGAGTGTAAGCAGGCTGCCAAGCAAGTCGTCGACGTTTATATGGGCGAGCAGAAGAAGGCCATCGATAGCCTGAAAGCCCAGGAAGCTAAGCATAAGGCCAAGGTGCAGAAGGCCCAGGAAGAGGAGAGAATGAAGAAGAATCAGGAGAAAAAGGAGAAGGCGGCGGCTAAAAAGGGTACATTTGCAGGAAAAAAAACTATAGAAATATTCTGGAATTTGAGATATCAGAGCCCGTTTTCCATTAATAACGCATTTTCAGAATTTCCGGAAATTCTGCAGTTTCCTGGATGATTTTCTCAATTTTACAGGAAAAAATGATCAAATTTTGACATTTTCTTTAAATATGGCGAGATAACTAGCCATAATATAATTGTTAAAAAATTAGTTAAATTTTGCAAAAATTACATAATCATCCGTTTTTTCTTTAACATGGTAAGAAATCTGGCTATTCTGGGAGAAATTCACTTTAAAAATACCAAAATTGGCTTAAAAATCGATAGTTCAACCGAAAACTGTCAGAAAAGTACTTTTAAAAGTGACACAAAAACTGAGAATTTTCAATTAATATATGTATTTGTATATTAATTGAAATTTCTCAGCTTTTGTGTCATTTTTCTGGCAGTTTTCGGTGGAATTATCGATTTTTAAGCCAATTTTGATTTTTTTAAAGGCAAATTTCCTCCAGACTGGCTAGTTT
