# Supplementary figures and images for: Characterization of Microsporidia-Induced Developmental Arrest and a Transmembrane Leucine-Rich Repeat Protein in Caenorhabditis elegans
Source: PLoS One. 2015 Apr 13;10(4):e0124065. doi: 10.1371/journal.pone.0124065 (PMC4395247; doi:10.1371/journal.pone.0124065)

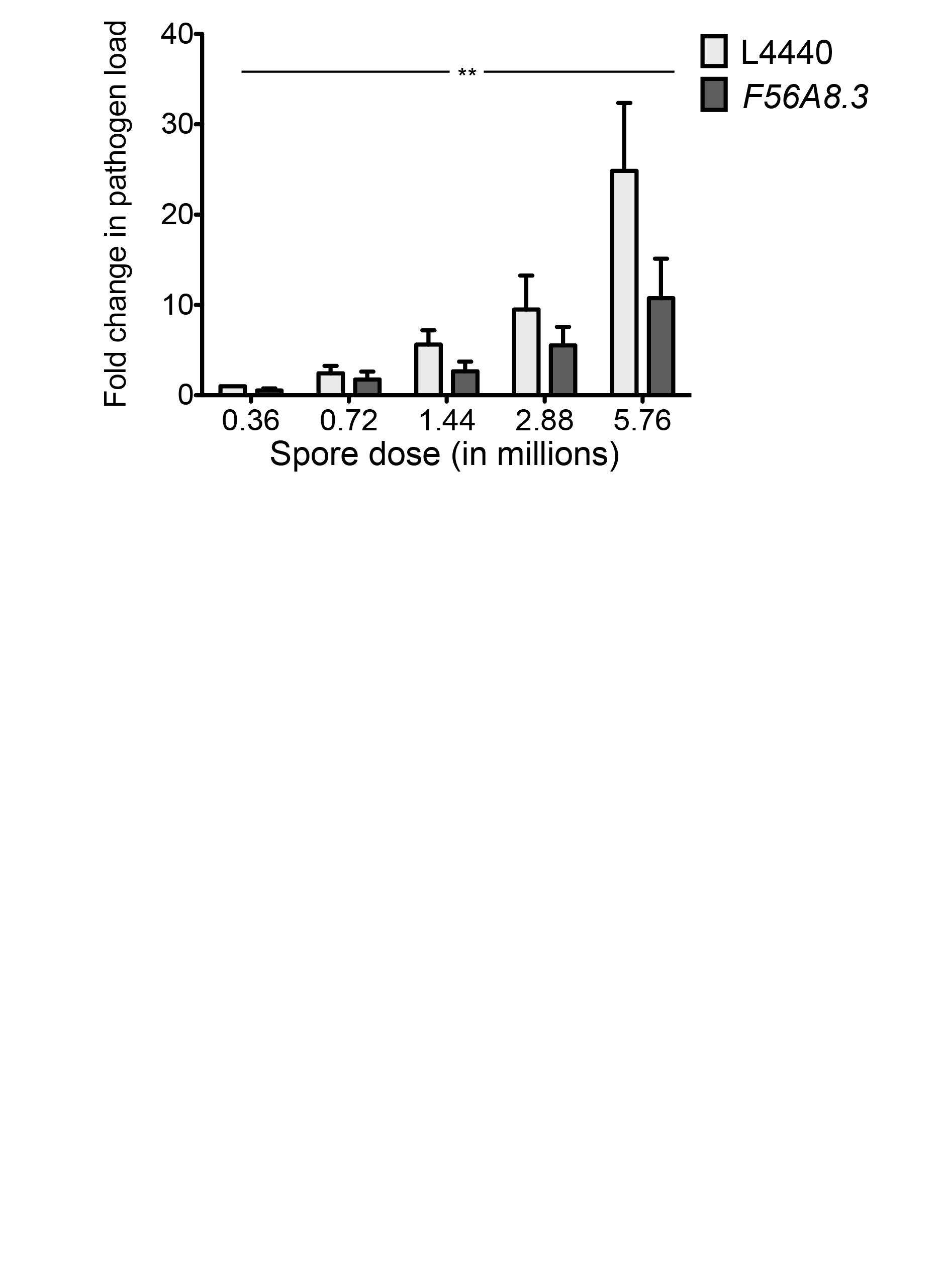

Supplement: S1 Fig — Pathogen load at 30 hpi on control or F56A8.3 RNAi measured as the fold change in N. parisii β-tubulin transcript by qRT-PCR relative to L4440 infected at the lowest dose. Animals were infected at L2/L3 stage. Data are represented as mean values with SEM from three independent experiments (**p = 0.0022, two-way analysis of variation, testing RNAi treatment effecting pathogen load at all doses). (TIF) [file pone.0124065.s002.tif]

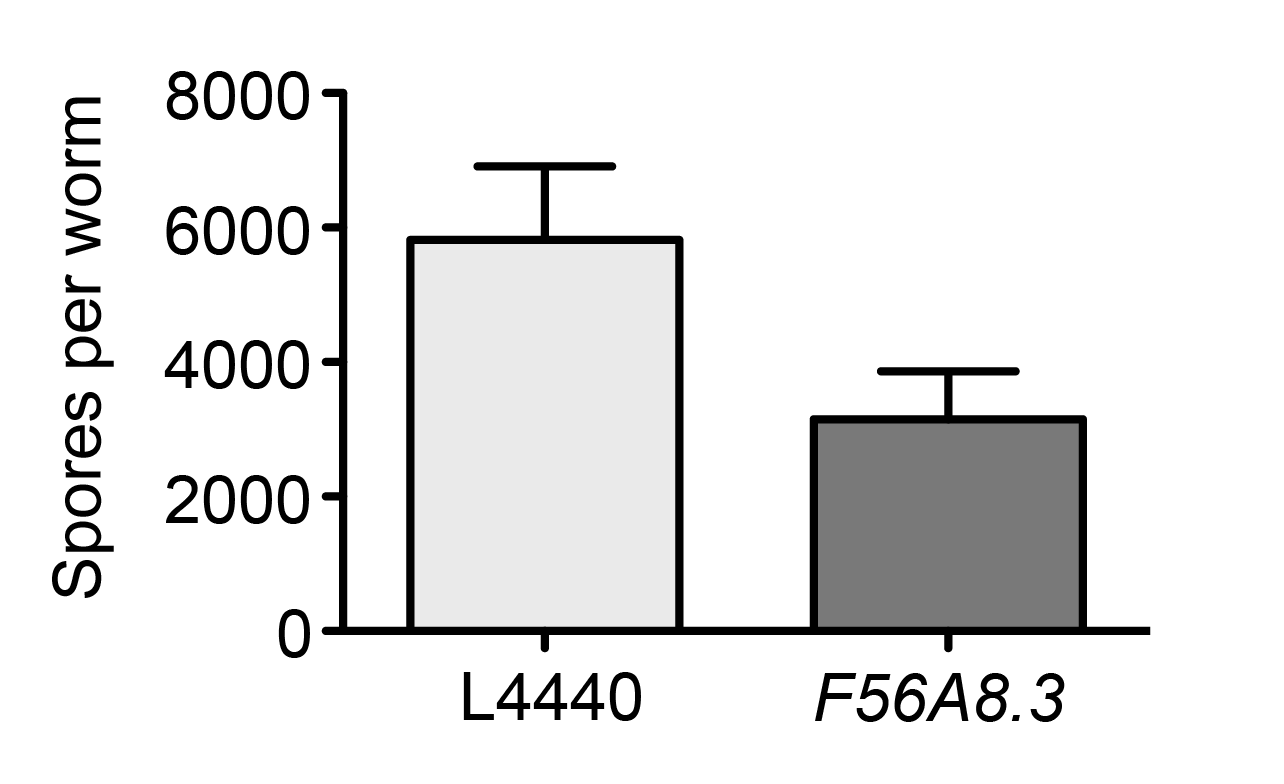

Supplement: S2 Fig — Pathogen load at 40 hpi with C. elegans infected at the L1 stage on control or F56A8.3 RNAi measured as the average number of spores produced per animal. Data are represented as mean values with SEM from two independent experiments. (TIF) [file pone.0124065.s003.tif]

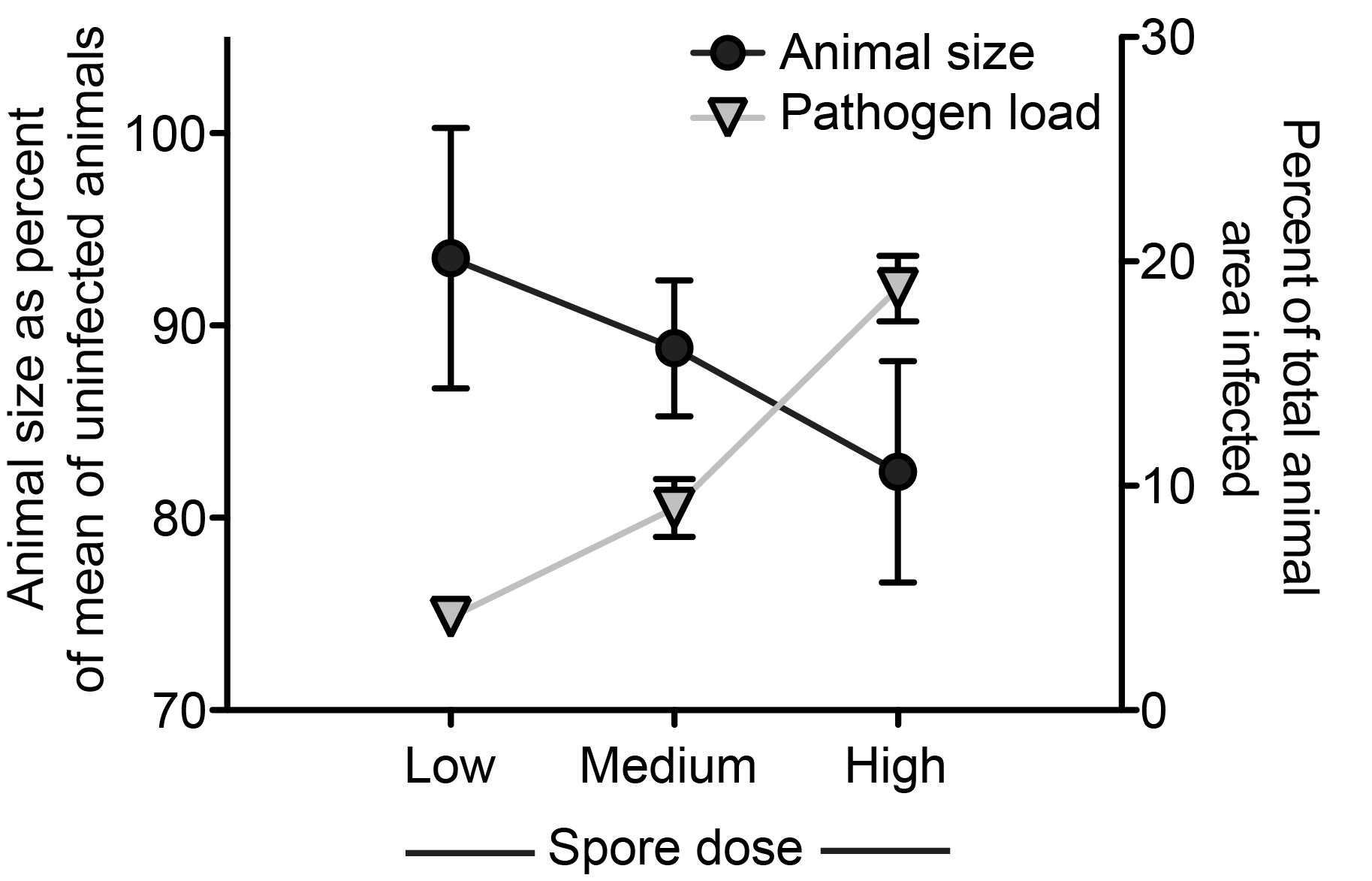

Supplement: S3 Fig — Animals were plated on L4440 bacteria for 18 hours and then infected for 24 hours using a low (3.63 x 10^5^ spores), medium (1.45 x 10^6^ spores), or high dose (5.80 x 10^6^ spores) of N. parisii spores on a 10 cm RNAi plate. Pathogen load was measured by FISH to N. parisii 18s rRNA and the percent area of the animal infected was calculated using ImageJ. Animal size was calculated by ImageJ and presented as the percent of the mean size of uninfected animals conducted in parallel. Data are represented as mean values with SEM of 20 individual animals in a single experiment. (TIF) [file pone.0124065.s004.tif]

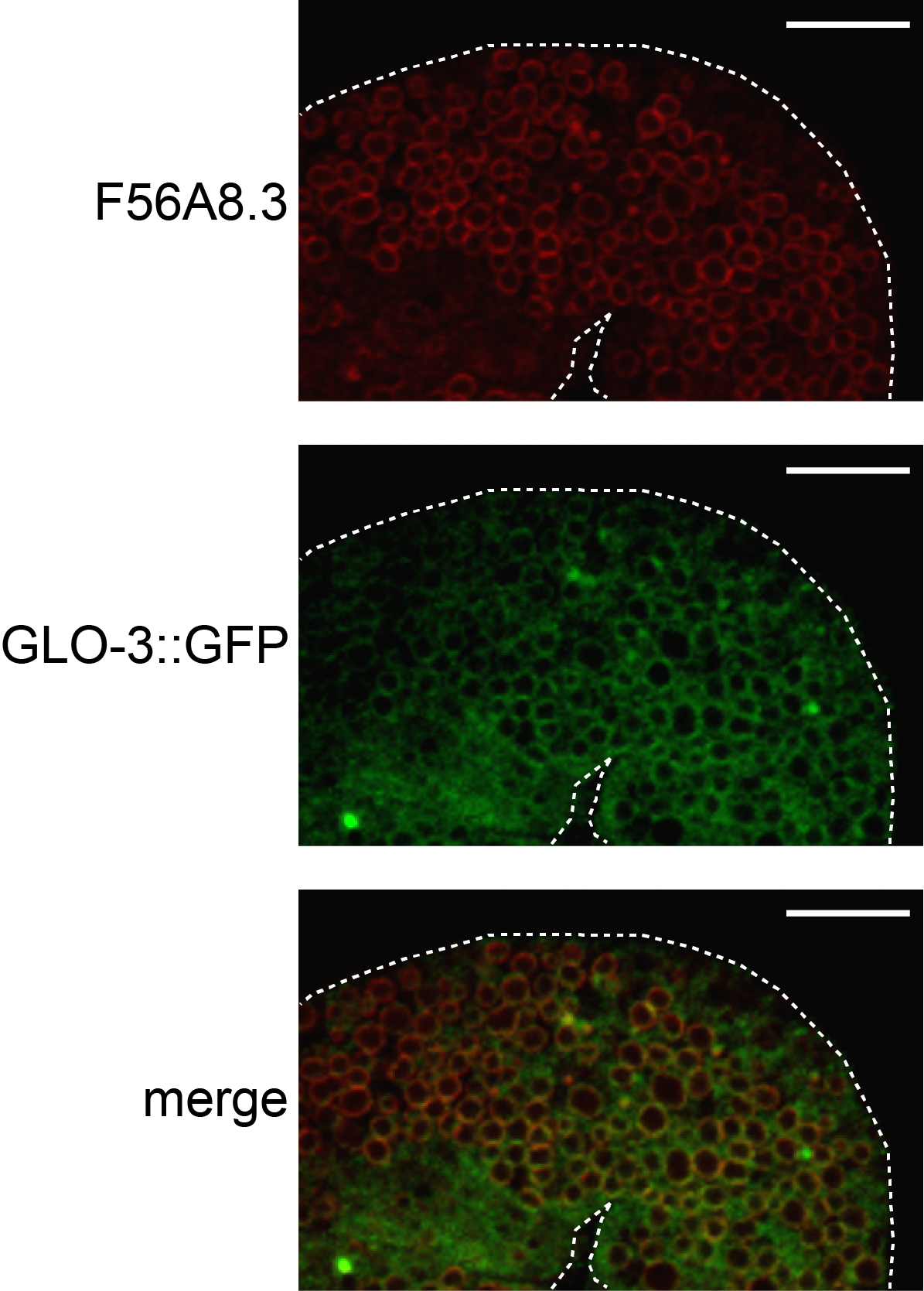

Supplement: S4 Fig — Representative image of endogenous F56A8.3 colocalization relative to GLO-3::GFP in the GH351 transgenic strain Scale bar = 10 μm. (TIF) [file pone.0124065.s005.tif]

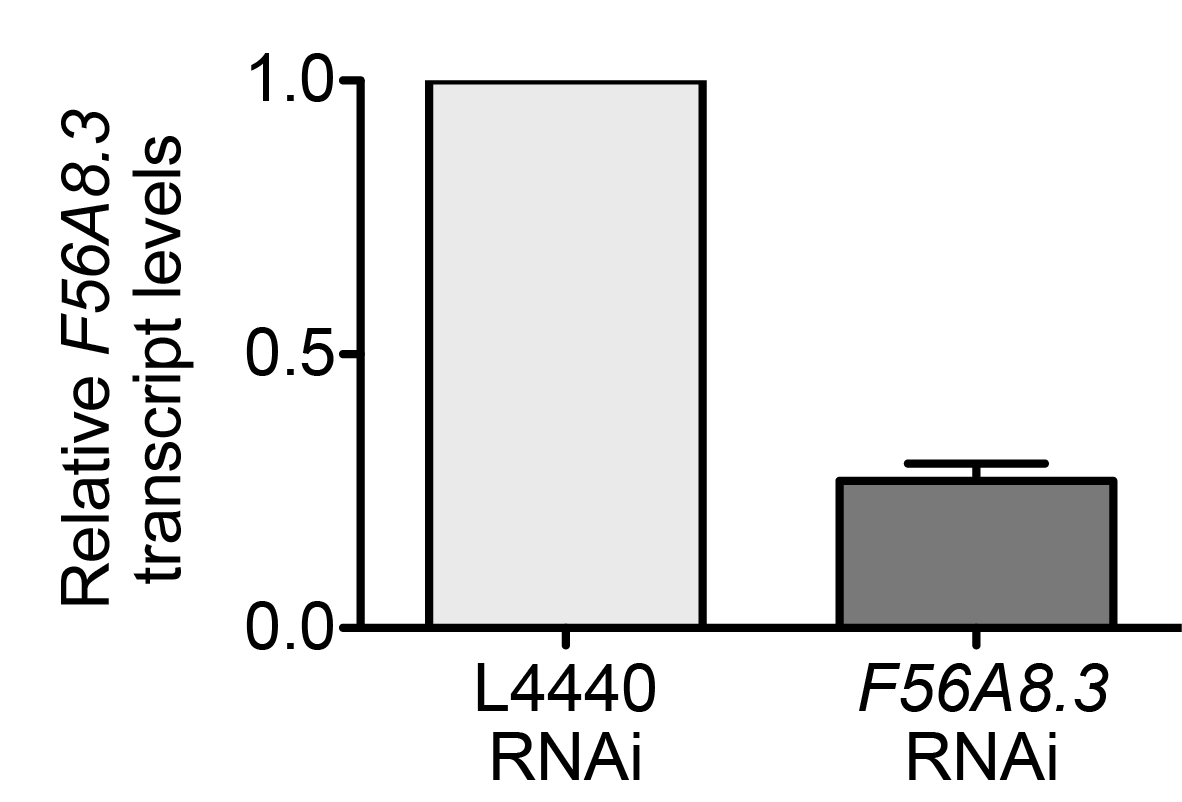

Supplement: S5 Fig — qRT-PCR analysis of the amount of F56A8.3 transcript in C. elegans grown on control or F56A8.3 RNAi measured as the fold change relative to L4440. Transcript levels were normalized to snb-1. Data are represented as mean values with SEM from two independent experiments. (TIF) [file pone.0124065.s006.tif]
